# Laparoscopic gastrojejunostomy with laparoscopic-assisted percutaneous endoscopic gastrostomy for superior mesenteric artery syndrome with dysphagia: a case report

**DOI:** 10.1186/s40792-022-01522-6

**Published:** 2022-09-01

**Authors:** Akiharu Kimura, Nobuhiro Morinaga, Wataru Wada, Kyoichi Ogata, Akiko Morishita, Takayuki Okuyama, Hiroyuki Kato, Makoto Sohda, Ken Shirabe, Hiroshi Saeki

**Affiliations:** 1 Department of Surgery, Kiryu Kosei General Hospital, 6-3 Orihime-Cho, Kiryu, Gunma 376-0024 Japan; 2 Department of General Surgical Science, Graduate School of Medicine, Gunma University, 3-39-22 Showa-Machi, Maebashi, Gunma 371-8511 Japan

**Keywords:** Superior mesenteric artery syndrome, Dysphagia, Laparoscopic gastrojejunostomy, Laparoscopic-assisted percutaneous endoscopic gastrostomy

## Abstract

**Background:**

Superior mesenteric artery (SMA) syndrome denotes a mechanical duodenal obstruction between the SMA and aorta. Total parenteral or enteral nutrition is the treatment of choice. However, surgical intervention is indicated if the patient’s condition does not improve with conservative treatment. Here, we describe a case of SMA syndrome with dysphagia treated by laparoscopic gastrojejunostomy with laparoscopic-assisted percutaneous endoscopic gastrostomy.

**Case presentation:**

A 64-year-old man was admitted to another hospital because of appetite loss and vomiting. There, he was diagnosed as having superior mesenteric artery (SMA) syndrome after appropriate investigation. He had had a cerebral infarction at age 57 years, since which he had lived in social housing because of complications of that infarction. A nasogastric tube was inserted into the third portion of the duodenum beyond the constricted section. He was discharged 2 months after admission his condition having improved. He was subsequently referred to our hospital for gastrostomy because the nasogastric tube had been in place for a long time and his condition had not improved. Additionally, gastrostomy was needed as a route for enteral nutrition because he had dysphagia, which had persisted despite attempts at rehabilitation, restricting his food intake to small amounts. Computed tomography (CT) revealed compression of the third portion of the duodenum between the SMA and aorta. After obtaining informed consent, we planned an operative procedure. We performed laparoscopic gastrojejunostomy under general anesthesia, followed by laparoscopic-assisted percutaneous endoscopic gastrostomy. The operation time was 156 min and there was little blood loss. Contrast radiography on postoperative day 3 revealed no evidence of leakage or stenosis. Enteral nutrition via the gastrostomy was started. He was discharged from our hospital on the 27th postoperative day. The gastrostomy was well tolerated and there has been no evidence of recurrence of SMA syndrome during follow-up.

**Conclusion:**

Gastrostomy is often performed to provide a route for administering enteral nutrition in patients with dysphagia. Development of SMA syndrome in patients with dysphagia necessitates operative management of the obstruction. Here, we describe a case of SMA syndrome with dysphagia treated by laparoscopic gastrojejunostomy with laparoscopic-assisted percutaneous endoscopic gastrostomy.

## Background

The term superior mesenteric artery (SMA) syndrome denotes a mechanical obstruction of the duodenum between the SMA and aorta. If the patient’s condition does not improve with conservative treatment, surgical intervention is needed. Here, we describe a case of SMA syndrome with dysphagia treated by laparoscopic gastrojejunostomy with laparoscopic-assisted percutaneous endoscopic gastrostomy.

## Case presentation

**Fig. 1:**
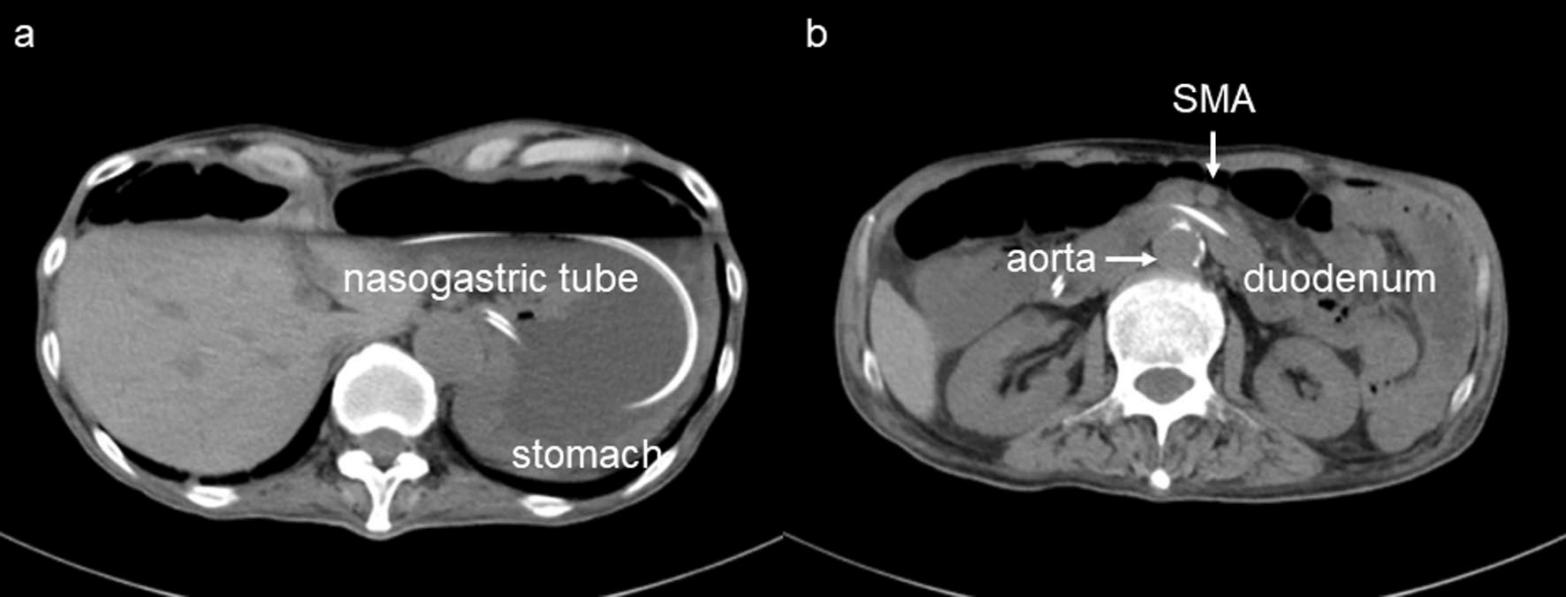
Abdominal computed tomography findings. **a** The stomach is dilated because of obstruction of the duodenum. **b** The third portion of the duodenum is compressed between the SMA and aorta

**Fig. 2:**
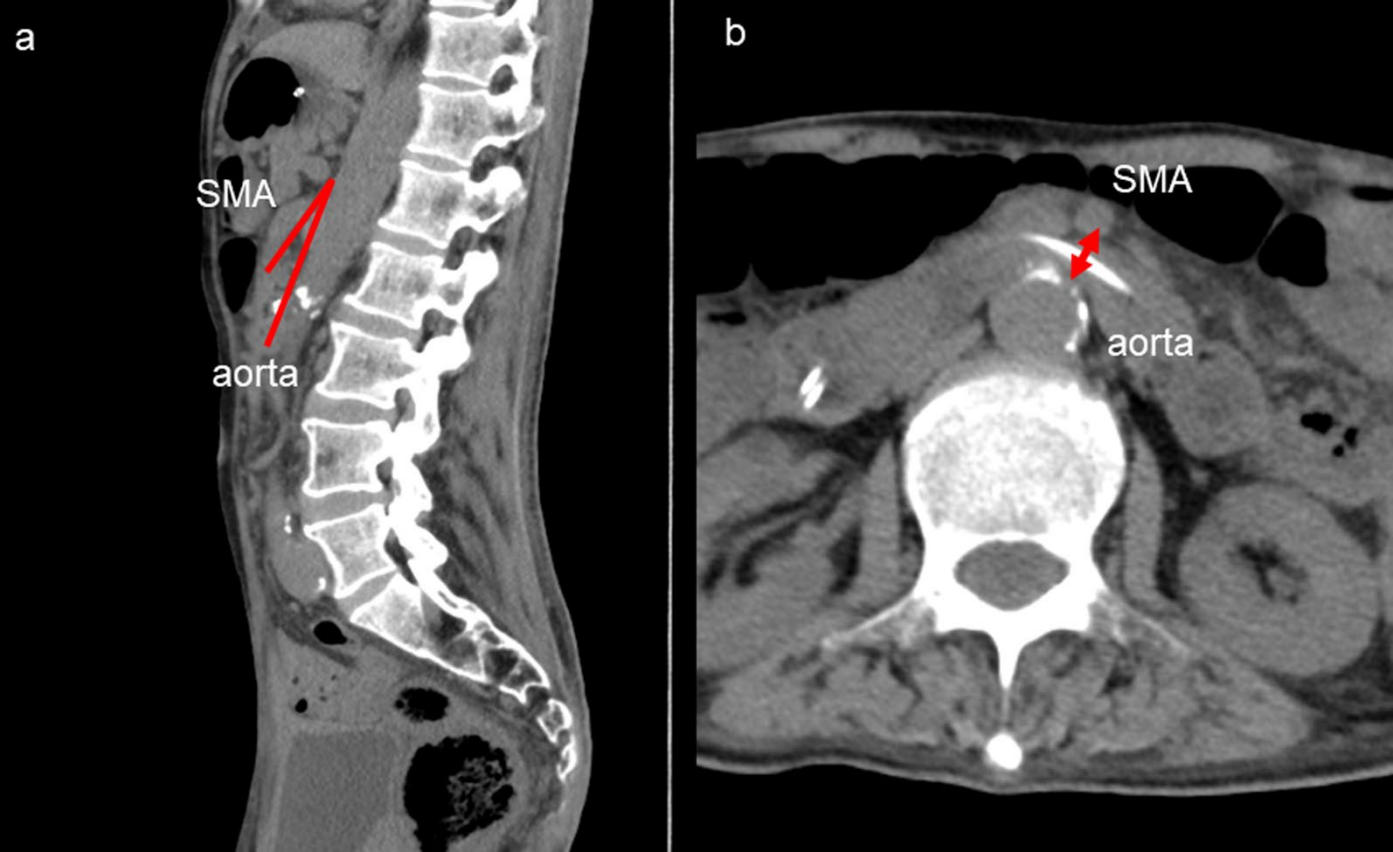
Computed tomography findings. **a** Sagittal view. **b** Axial view. The aorto-mesenteric angle is narrow at 18° and the aorto-mesenteric distance short at 8 mm

**Fig. 3:**
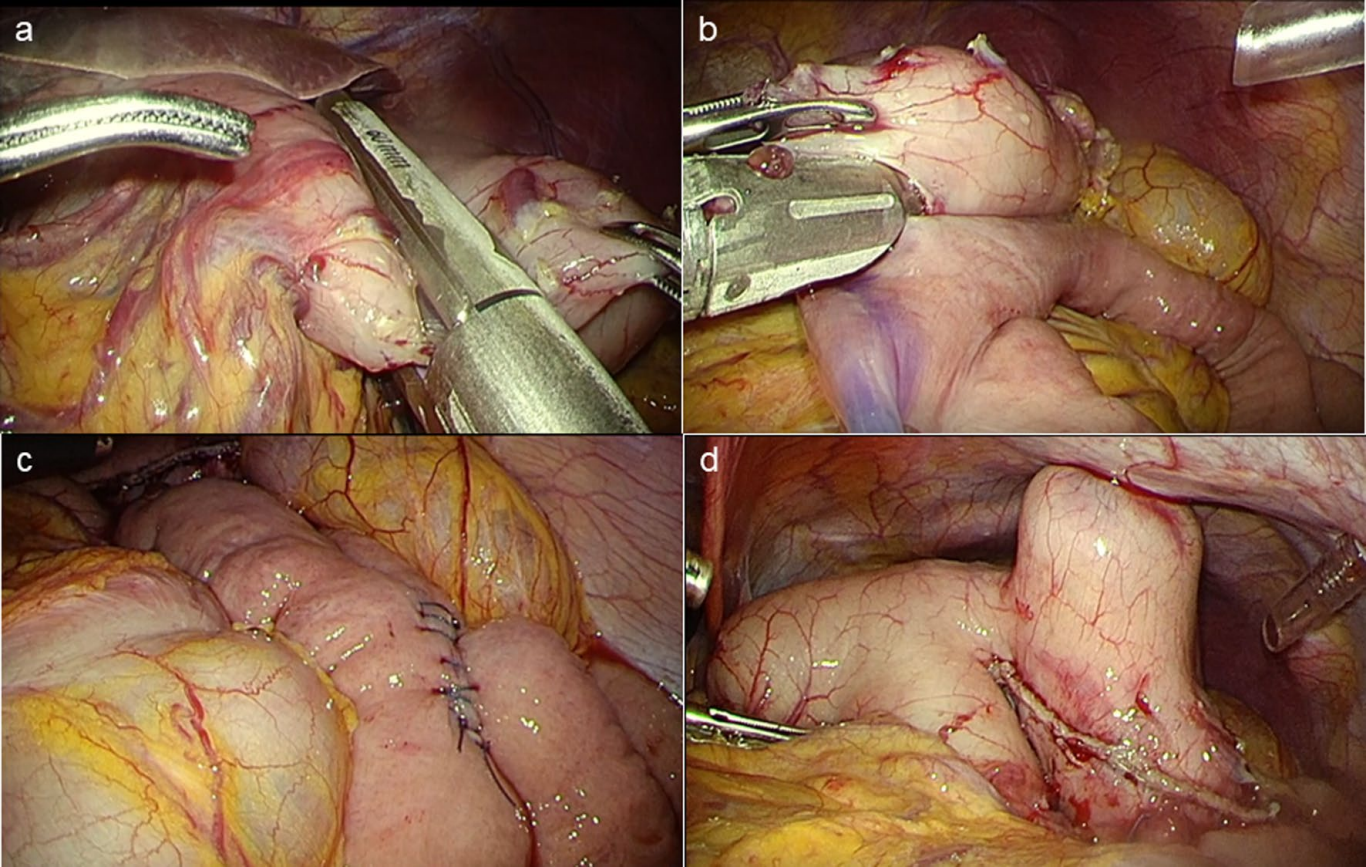
Operative findings. **a** The greater curvature was transected with a liner stapler to increase flow to the jejunal bypass. **b** A 60-mm Endo GIA Tri-Staple™ purple cartridge (Medtronic, Minneapolis, MN, USA) was inserted into the small incisions in the jejunum and stomach to create a gastrojejunostomy. **c** A Braun anastomosis was created to prevent afferent loop syndrome. **d** Laparoscopic-assisted percutaneous endoscopic gastrostomy was also performed, the gastrostomy being located on the oral side of the gastrojejunostomy

**Fig. 4:**
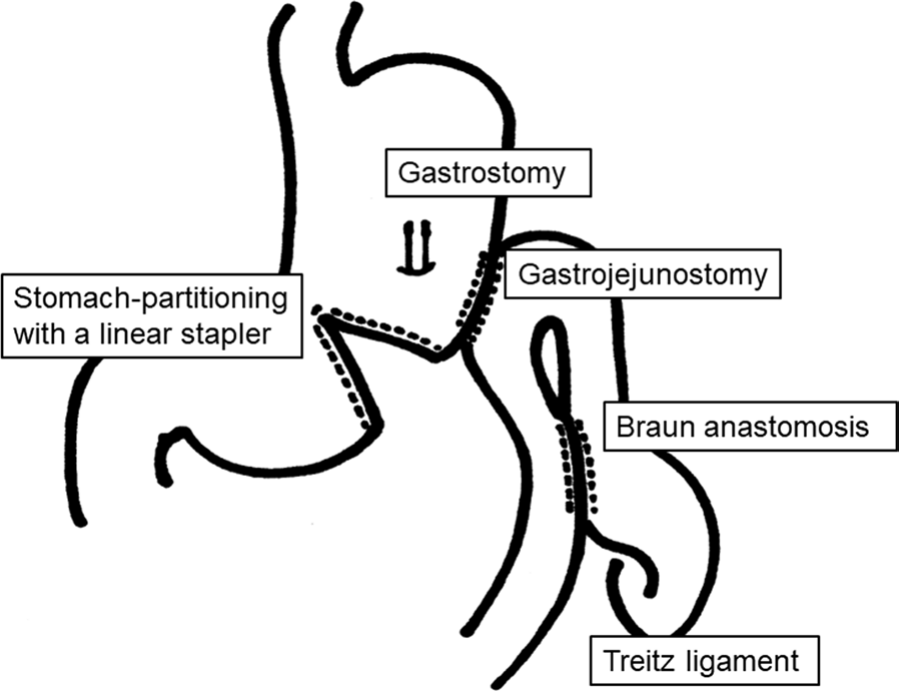
Schematic figure of the operation. The greater curvature of the stomach was transected with a linear stapler to increase flow to the jejunal bypass (stomach-partitioning). The gastrostomy was placed on the oral side of the gastrojejunostomy

**Fig. 5:**
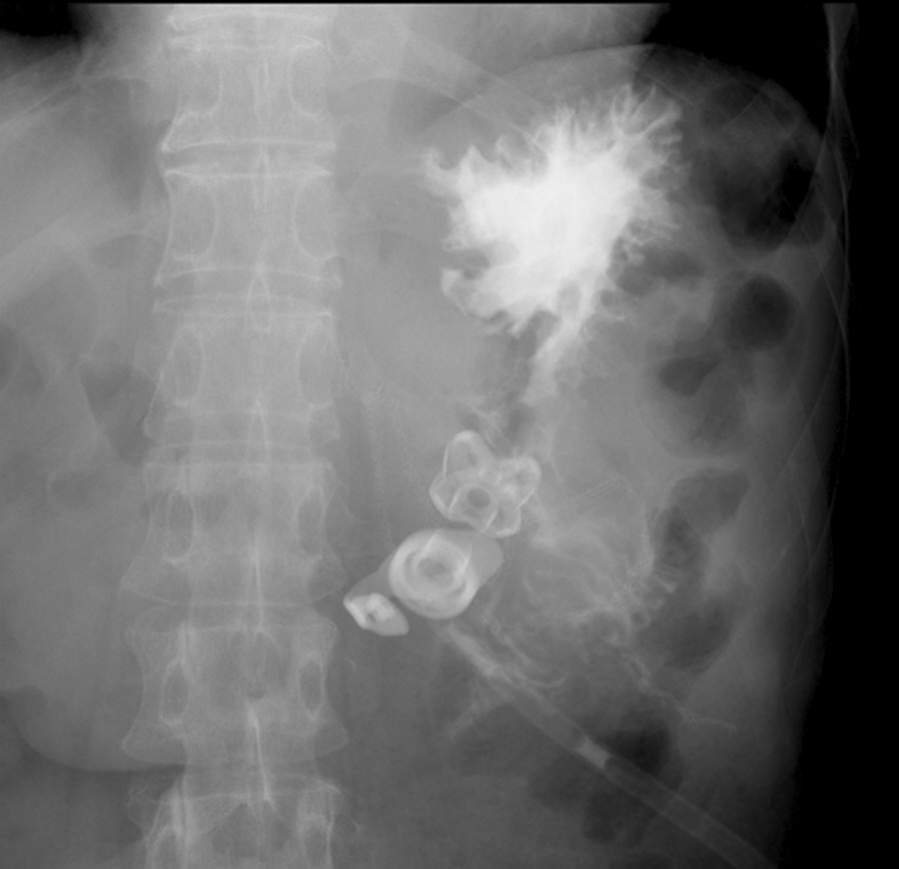
Contrast radiography findings. There was no evidence of postoperative leakage or stenosis

A 64-year-old man was admitted to another hospital because of appetite loss and vomiting. He was diagnosed as having superior mesenteric artery (SMA) syndrome after appropriate investigations. He had been living in social housing because of complications of a cerebral infarction he had had at the age of 57 years. A nasogastric tube was inserted into the third portion of the duodenum beyond the constriction. He was discharged 2 months after admission, his condition having improved. He was subsequently referred to our hospital for gastrostomy because the nasogastric tube had been in place for 3 months. Computed tomography (CT) revealed compression of the third portion of the duodenum between the SMA and aorta (Fig. 1). The aorto-mesenteric angle was narrow at 18° and the aorto-mesenteric distance short at 8 mm (Fig. 2). His stomach and duodenum were distended, consistent with persistent SMA syndrome. Surgical intervention for SMA syndrome was needed because his condition had not improved. An additional indication for gastrostomy was to provide a route for enteral nutrition because he had dysphagia. After obtaining informed consent, a surgery was planned. His body height was 165.0 cm, his weight 42.0 kg, and his body mass index (BMI) 15.43. His abdomen was soft and not tender. Laboratory tests showed white blood cell count 6.8 × 10^9^/L, red blood cell count 4.84 × 10^12^/L, hemoglobin concentration 14.3 g/dL, total protein concentration 5.6 g/dL, albumin concentration 2.9 g/dL, and cholinesterase concentration 177 U/L. Laparoscopic gastrojejunostomy was performed under general anesthesia. First, a trans-umbilical incision was made and a 12-mm port inserted. After inducing a pneumoperitoneum, 5-mm ports were inserted in the right and left right upper quadrants and 12-mm ports in the right and left lateral abdomen. The omentum was dissected to open the omental bursa. The greater curvature of the stomach was transected with a linear stapler to increase flow to the jejunal bypass (Fig. 3a). Small incisions were made in the proximal jejunum (30 cm from the Treitz ligament) and posterior wall of the upper body of the stomach (oral side of the stapled line). A 60-mm Endo GIA Tri-Staple™ purple cartridge (Medtronic, Minneapolis, MN, USA) was then inserted into the small incisions in the jejunum and stomach to create a gastrojejunostomy (Fig. 3b). The common point of entry was closed by a hand-sewn continuous running suture using a 3–0 V-Loc™ (Medtronic), after which a Braun anastomosis was created to prevent afferent loop syndrome (Fig. 3c). Next, a laparoscopic-assisted percutaneous endoscopic gastrostomy was performed, the gastrostomy being on the oral side of the gastrojejunostomy (Fig. 3d, Fig. 4). The operation time was 156 min and there was little blood loss. Contrast radiography on postoperative day 3 revealed no evidence of leakage or stenosis (Fig. 5). Enteral nutrition via the gastrostomy was started and attempts at dysphagia rehabilitation continued. However, his ability to swallow did not improve and he was able to eat only small amounts of food, necessitating enteral nutrition via the gastrostomy. He was discharged from our hospital on the 27th postoperative day. Laboratory tests before discharge showed total protein concentration 5.8 g/dL, albumin concentration 3.0 g/dL and his nutritional condition was slightly improved. We perform the exchange of the gastrostomy every 6 months and there is no evidence of gastric dilatation on the radiographic image. There have since been no problems with the gastrostomy or evidence of recurrence of SMA syndrome.

## Discussion

The term SMA syndrome denotes a mechanical obstruction of the duodenum between the SMA and aorta. This syndrome can result in symptoms such as epigastralgia, abdominal distension, and vomiting of bile as a result of compression of the third portion of the duodenum between the SMA and aorta [1–3]. Characteristic manifestations include rapid weight loss, kyphosis, and abdominal aortic aneurysms. These patients may be bedridden for long periods, necessitating surgery [4–6]. Radiologic studies such as upper gastrointestinal barium study and abdomen CT are useful for diagnosing SMA syndrome [7]. CT imaging shows a mechanical obstruction of the duodenum between the SMA and aorta. The angle between the aorta and SMA is less than 25° and aorto-mesenteric distance less than 8 mm [8, 9]. Our patient met these criteria.

The treatment of choice for SMA syndrome is conservative, with total parenteral or enteral nutrition. This approach can promote an increase in the amount of fat in the retroperitoneum and mesentery. However, surgical intervention is needed if the patient’s condition does not improve with, or SMA syndrome recurs after, conservative treatment. Duodenojejunostomy, gastrojejunostomy, duodenum mobilization, and anterior transposition of the duodenum are recognized operative treatments for SMA syndrome. Recently, laparoscopic gastrojejunostomy or duodenojejunostomy has been performed on patients with SMA syndrome. In 1998, Gersin and Heniford reported the first laparoscopic duodenojejunostomy for SMA syndrome [10]. Currently, the most commonly performed laparoscopic procedure for SMA syndrome is laparoscopic duodenojejunostomy [11], whereas laparoscopic gastrojejunostomy is performed on patients with advanced gastric cancer and pyloric stenosis [12]. Because we have considerable experience of laparoscopic gastrojejunostomy for unresectable advanced gastric cancer, we elected to perform a laparoscopic gastrojejunostomy in the present case. Reduced port or single-incision laparoscopic surgery (SILS) has been adopted for management of SMA syndrome [7, 13]. However, the indications for SILS are controversial. Some studies have failed to demonstrate that SILS is superior to a conventional laparoscopic approach [14, 15]. In contrast, there have been several reports of favorable outcomes with SILS [16–19]. In the present case, we performed conventional laparoscopic surgery with five ports because we needed to close the common point of entry of the gastrojejunostomy and Braun anastomosis with a hand-sewn continuous running suture.

Additionally, we performed laparoscopic-assisted percutaneous endoscopic gastrostomy after creating the gastrojejunostomy. Laparoscopic-assisted percutaneous endoscopic gastrostomy is useful in minimizing inadvertent puncture of other organs and dealing with internal and external bleeding from the stomach. Moreover, this approach results in less postoperative pain and wound infection than does open gastrostomy. It also provides a satisfactory visual field during the procedure. Carlos et al. reported performing laparoscopic–percutaneous combined gastrostomy on 17 patients [20]. They found that this procedure is fast, feasible, safe, and cost effective. A laparoscopic-assisted approach enables safe construction of a percutaneous endoscopic gastrostomy after gastrojejunostomy.

## Conclusions

We here present a case of SMA syndrome with dysphagia treated by laparoscopic gastrojejunostomy with laparoscopic-assisted percutaneous endoscopic gastrostomy. A laparoscopic approach can reduce operative stress and promote postoperative nutrition management and rehabilitation.

## Data Availability

All data generated or analyzed during this study are included in the published article.

## Abbreviations

BMI: Body mass index
CT: Computed tomography
SILS: Single-incision laparoscopic surgery
SMA: Superior mesenteric artery
